# Maternal emotional distress, caregiving burden, and service access among mothers of children with autism spectrum disorder: a qualitative study

**DOI:** 10.3389/fpsyt.2026.1971477

**Published:** 2026-09-25

**Authors:** Hawa Alabdulaziz, Salmah Alghamdi, Khulood S. Alharbi, Huda Rubayan M. Alrashdi, Johara Sohail Al-mutairi, Fatmah H. Aljuhani

**Affiliations:** 1 King Abdulaziz University, Jeddah, Saudi Arabia; 2 King Salman Medical City, Madinah, Saudi Arabia; 3 Khaybar General Hospital, Madinah, Saudi Arabia; 4 Yanbu General Hospital, Yanbu, Saudi Arabia

**Keywords:** autism spectrum disorder, caregiving burden, family support, maternal mental health, public mental health, Saudi Arabia, service access, stigma

## Abstract

**Introduction:**

Mothers raising children with autism spectrum disorder (ASD) may experience substantial emotional distress and caregiving burden, whilst family support andaccess to services can shape how these demands are managed. Yet in-depthqualitative evidence on these public mental health dimensions remains limited inSaudi Arabia. This study explored mothers’ experiences of raising a child diagnosedwith ASD.

**Methods:**

Using a qualitative descriptive design informed by a contextualistposition, the study recruited ten mothers through a specialist autism centre inMadinah and the surrounding community. Trained female researchers, all registered nurses, conducted semi-structured interviews in Arabic, each lastingapproximately 60 to 90 minutes. Recordings were transcribed verbatim inArabic; a professional translator produced English transcripts, which a secondbilingual researcher checked before reflexive thematic analysis.

**Results and discussion:**

Seven interrelated themes captured the mothers’ accounts: diagnosis as an emotional rupture; disruption of daily life by behavioural challenges; learning to understand the child across communication barriers; the cumulative toll of continuous care; adaptation through acceptance, patience, and faith; family support as a buffer or added burden; and compensating for uneven service experiences. Mothersdescribed substantial emotional and caregiving demands, but their experiences varied according to family support and access to services. One account of comprehensive state support provided an important counter-case to the morecommon experience of costly or fragmented provision. The findings position maternal emotional distress and caregiving burden as public mental health concerns shaped by family, social, and service contexts. They support a public mental health response that links maternal mental health promotion with accessible, coordinated, and family-centred care.

## Introduction

Autism spectrum disorder (ASD) is a neurodevelopmental condition characterised by persistent difficulties in social communication and interaction, alongside restricted or repetitive patterns of behaviour, interests, or activities (1). Epidemiological studies report substantial variation in prevalence across settings. Salari et al. (2) estimated a global prevalence of about 0.6%, whilst Akomolafe et al. (3) reported marked methodological heterogeneity across the Middle East and North Africa, with evidence suggesting a possible increase over time. Saudi estimates also vary by study design and setting. Alsulami et al. (4) reported a pooled prevalence of approximately 1.3%, AlBatti et al. (5) found a prevalence close to 2.5% amongst toddlers in Riyadh, and Sabbagh et al. (6) reported lower centre-based population estimates in Makkah and Jeddah. More recently, Khamees et al. (7) highlighted discrepancies across available Saudi data sources and the continuing uncertainty around national prevalence. Overall, these findings identify ASD as an important public health concern for Saudi families.

An ASD diagnosis can affect the wider family as well as the child, and parents may experience substantial psychological demands. Studies across Arab settings report elevated anxiety, depression, and burnout amongst parents of children with ASD, together with lower quality of life than amongst parents of typically developing children (8, 9). In Saudi Arabia, Alenezi et al. (10) documented substantial psychological strain amongst parents, whilst AlHefdhi et al. (11) reported an association between experienced discrimination and poorer parental quality of life. Alicka et al. (12) likewise identified high levels of stress, anxiety, and depression across the parental literature. Studies in other settings report a similar pattern (13, 14).

Research also points to a gap between early parental concern and formal diagnosis. Parents often notice developmental differences within the first two years of life, whilst diagnosis may not occur until three to five years of age (15, 16). Jayanath and Ozonoff (17) reported a similar pattern in Malaysia. Within Saudi Arabia, studies continue to identify gaps in autism-related services and support for families (18, 19). Because nurses frequently serve as a first point of contact, the autism-related knowledge gaps reported by Albloushi et al. (20) have particular relevance. Abualhommos et al. (21) reported moderate-to-high overall community knowledge of ASD whilst identifying gaps in understanding specific aspects of the condition.

Cultural and service contexts further shape how families experience ASD. Extended-family networks may provide practical and emotional support, but different expectations can also create pressure or misunderstanding (9). Stigma remains a prominent concern. Sulaimani and Mursi (22) documented stigma experienced by Saudi mothers raising children with ASD, whilst studies elsewhere in the region report related experiences (23, 24). Reviews and qualitative studies also identify religious coping as a prominent strategy in Arab and Muslim contexts (8, 25). Salami and Alhalal (26) identified perceived social support and family functioning amongst factors associated with parental quality of life. Their study also found that mothers reported lower quality of life and greater reliance on emotion-focused coping than fathers. Meanwhile, families continue to report concerns about the availability, affordability, and coordination of services (18, 19, 27).

Despite this growing evidence base, few in-depth qualitative studies in Saudi Arabia have examined diagnosis, everyday caregiving, adaptation, family support, stigma, and service navigation as connected dimensions of maternal wellbeing. This gap is relevant to public mental health because maternal distress and adaptation develop within interacting risk and protective contexts, including caregiving demands, family support, social responses, and access to care. Qualitative studies from Jordan, the United Arab Emirates, Iran, and Greece have begun to illuminate related experiences (23, 24, 28, 29), whilst Makino et al. (30) found that much of the literature on parental experiences of ASD diagnosis still comes from high-income Western settings. The present study addresses this gap by exploring the experiences of ten mothers raising a child with ASD in Madinah. It examines the emotional, behavioural, and social challenges they encounter, the ways they adapt, and the family and service supports on which they rely. By locating these accounts within Madinah’s cultural and health-service context, the study seeks to inform maternal mental health support, culturally responsive service design, stigma reduction, and policy relevant to Saudi Arabia’s Vision 2030 (31).

### Study aim and objectives

The aim of this study was to explore mothers’ experiences of raising a child diagnosed with ASD. The study addressed three objectives:

- To explore mothers’ experiences of everyday life whilst raising a child with ASD.
- To examine the challenges mothers encounter in caring for a child with ASD.
- To explore mothers’ experiences of, and satisfaction with, the resources and treatment available for ASD.

## Materials and methods

### Study design and epistemology

This study used a qualitative descriptive design informed by a contextualist position. The research team treated participants’ accounts as meaningful descriptions of experience whilst recognising that cultural, familial, and service contexts shaped those accounts. The team used reflexive thematic analysis (32), which treats researcher interpretation as part of analysis rather than a bias that can be removed. This combination suited an applied study seeking a contextualised account of mothers’ experiences. The qualitative descriptive design provided the overall applied methodological orientation, whilst the team used reflexive thematic analysis as the analytic approach to interpret patterns across participants’ accounts. The Consolidated Criteria for Reporting Qualitative Research (COREQ) guided reporting (33). A separate COREQ checklist accompanies the manuscript.

### Sample and setting

A specialist autism centre in Madinah, Saudi Arabia, used a purposive recruitment approach by circulating the study invitation to mothers of children diagnosed with ASD. Ten mothers chose to participate. Recruitment also reached the wider community in Madinah, so participation was not confined to families attending the centre. The team did not organise recruitment around a numerical saturation threshold, which would sit uneasily with reflexive thematic analysis (34). Instead, the team used information power as a rationale for sample adequacy, considering the focused study aim, the specificity of the participant group, the depth of the interviews, and the analytic strategy (35). The centre circulated a Google Forms link containing study information and an informed-consent form. Participants differed in where they obtained autism-related services: two were receiving care at the centre, one was a physician employed there, and the remaining seven accessed services elsewhere. The centre therefore acted mainly as a recruitment route rather than as the shared source of care. **Table 1** summarises participant and child characteristics. [Institution name blinded for peer review.] Recruitment through one centre and the surrounding community, together with the small sample and single-city setting, limits the sociocultural range represented; participants nonetheless differed in where they accessed services, which added some variation to their service-related accounts.

**Table 1:** Participant and child characteristics.

Participant	Child sex	Child age (yrs)	Age at first concern	Age at diagnosis	Time since diagnosis	Reported severity
P1	Female	5–7	< 1 yr	4–5 yr	1–3 yr	Severe
P2	Male	5–7	1–2 yr	approx. 5 yr	1–3 yr	Mild
P3	Male	5–7	1–2 yr	approx. 2 yr	1–3 yr	Not specified
P4	Female	8–11	approx. 1 yr	1–2 yr	> 6 yr	Not specified
P5	Male	2–4	1–2 yr	approx. 2 yr	1–3 yr	Severe
P6	Female	5–7	approx. 2 yr	3 yr	1–3 yr	Moderate
P7	Male	2–4	1–2 yr	Early childhood	1–3 yr	Not specified
P8	Male	8–11	Toddlerhood	6–8 yr	< 3 yr	Mild
P9	Male	2–4	< 1 yr	2–3 yr	< 1 yr	Not specified
P10	Male	12–15	< 1 yr	4 yr	> 6 yr	Not specified

Participant identifiers are study codes. To protect confidentiality, the table groups children’s ages into bands, and the authors omitted names, institutions, diagnostic scores, and other potentially identifying details. Cell values reproduce information that mothers recounted during interviews, including clinician-reported details. The sample comprised 10 mothers; their children included 7 boys and 3 girls, aged 2 to 15 years, with time since diagnosis ranging from under 1 year to more than 6 years. Mothers reported co-occurring attention or hyperactivity difficulties in a minority of cases.

### Data collection

The research team used semi-structured interviews to cover the core study topics whilst allowing the interviewer to follow issues that participants considered important. The team developed the interview guide from literature on parental experiences of ASD and piloted it to assess clarity, cultural fit, and topic coverage. Core prompts invited mothers to describe the diagnosis and its impact, challenging behaviours and their effects on everyday and family life, difficulties in understanding the child’s needs and emotions, the influence of caregiving on their wellbeing, the ways they had adapted, and the role of family and community support. Trained female researchers, all registered nurses and fluent in Arabic and English, conducted the interviews in Arabic. Each participant chose a private setting. Interviews lasted approximately 60 to 90 minutes, and the researcher audio-recorded them with explicit consent.

### Data analysis

The analysis began with verbatim Arabic transcripts of the audio recordings. A professional translator produced English transcripts, and a second bilingual researcher checked them against the Arabic transcripts to preserve meaning and nuance. The team discussed and resolved translation discrepancies. The researchers then followed the six phases of reflexive thematic analysis described by Braun and Clarke (32). They first familiarised themselves with the dataset through repeated reading and listening, then generated initial codes inductively across the interviews. The team did not treat coding as a reliability exercise. Instead, collaborative discussions examined alternative interpretations and refined the developing analysis. The researchers brought codes together into candidate themes, reviewed those themes against both coded extracts and the dataset as a whole, and then defined, named, and illustrated them with representative quotations. They retained and interpreted disconfirming accounts, including one mother who described comprehensive state funding and another who experienced relief rather than distress at diagnosis, rather than absorbing those accounts into the dominant pattern.

### Reflexivity and positionality

Consistent with reflexive thematic analysis, the researchers treated their own perspectives as part of the analytic process. The team comprised female nurse-researchers based in Saudi Arabia who were familiar with the local health system. All members of the research team were registered nurses, and the principal investigators had extensive professional and research experience with children who have special needs, including ASD, alongside expertise in nursing research and in qualitative methods such as reflexive thematic analysis. None of the researchers had a direct clinical or supervisory relationship with the participants, and none was involved in their routine care at the centre. Prior professional experience with autism may have shaped the team’s assumptions during data generation and interpretation; the researchers used ongoing self-reflection during interviewing and analysis to critically examine how these assumptions informed the developing interpretation. Their proximity to the context supported rapport and cultural interpretation, but it also created a risk of over-identifying with mothers’ accounts or treating maternal advocacy as universal. The team used reflexive logs to record assumptions, discussed alternative readings during analysis, and deliberately returned to accounts that did not fit the dominant pattern. They evaluated analytic quality against the principles of reflexive thematic analysis rather than inter-rater reliability (34).

### Trustworthiness

The team used several complementary strategies to strengthen trustworthiness. Sustained engagement with the data, peer debriefing within the research team, and member reflections on a summary of the themes supported credibility. The team maintained an audit trail that documented the movement from extracts to codes and themes, which strengthened transparency and dependability. Reflexive practices supported confirmability without relying on claims of researcher neutrality. Finally, the manuscript provides contextual detail about the Saudi setting so readers can judge the relevance of the findings to comparable contexts. The study does not claim statistical generalisation.

### Ethical considerations

Two institutional review boards in Saudi Arabia approved the study on 19 October 2025 and 5 May 2025; institution names and approval identifiers are blinded for peer review. The research team informed mothers about the study’s purpose, procedures, potential risks and benefits, and their right to withdraw at any time without penalty. Mothers participated voluntarily and provided informed consent before data collection. The team protected confidentiality by using study codes, removing identifying details, storing data securely, and limiting access to the research team. The team also assured participants that taking part, or choosing not to take part, would not affect any services that they or their children received.

## Results

The analysis drew on in-depth interviews with ten mothers of children with ASD. **Table 1** shows that mothers generally reported concerns before formal diagnosis, with intervals ranging from months to several years. Through reflexive thematic analysis, the team developed seven interrelated themes, which the following sections present with illustrative verbatim quotations. To match the study’s focus, the seven themes are grouped under three overarching domains, namely maternal emotional distress, caregiving burden, and service access, with adaptation and family support presented as cross-cutting influences that shaped all three. Each participant code maps to **Table 1**, so readers can trace the child’s sex, age band, reported severity, and time since diagnosis behind a given account. Unless a quotation is attributed to a single mother, coded extracts illustrate experiences that recurred across several participants, whilst accounts reported by only one mother are signposted as such.

### Domain 1: maternal emotional distress

#### Theme 1: diagnosis as an emotional rupture

Across the interviews, mothers described diagnosis as an emotional rupture. They spoke of shock, fear, sadness, anxiety and, for some, a sense of emotional collapse. Several recalled struggling to accept the diagnosis and described days of crying or persistent worry about the child’s future. The emotional impact continued beyond the diagnostic appointment and entered everyday caregiving. For many mothers, the loss they described concerned not the child, but the future they had imagined for the child and family. These accounts describe mothers’ self-reported emotional responses; they do not represent clinical assessment and should not be read as formal diagnoses of anxiety, depression, or related disorders.

“When I heard the diagnosis, I felt completely broken emotionally.” (P1; daughter aged 5–7 years, reported severe)

Mothers also conveyed disbelief and disorientation. Some reported sleeplessness and loss of appetite. Family members did not always carry the distress equally; in some accounts, one parent was coping with the diagnosis whilst also trying to steady the other.

“I was shocked and could not accept it at the beginning.” (P2) “I cried for days and felt lost.” (P3)

### Domain 2: caregiving burden

#### Theme 2: daily life disrupted by behavioural challenges and resistance to routine

Mothers described feeding refusal, resistance to training, limited engagement in play, and difficulty cooperating with therapy as recurring disruptions to daily life. These behaviours made ordinary activities harder to plan and sustain. Their unpredictability also disturbed the child’s routine and affected the wider household.

“My child refused to eat, drink, or cooperate with training.” (P1) “He did not follow any daily routine.” (P2)

Part of the strain came from a gap between knowing that consistency mattered and being unable to maintain it in practice. Some mothers linked this difficulty to guilt or a sense of inadequacy. Others avoided public outings because they feared meltdowns, and this gradually narrowed family social life. Analytically, these accounts suggest that behavioural unpredictability did more than complicate individual tasks: it destabilised the routines through which mothers organised family life. Ordinary activities became repeated negotiations between maintaining consistency, responding to the child’s tolerance, and managing mothers’ own feelings of adequacy.

“Simple activities became very difficult.” (P3) “Managing his behavior was exhausting.” (P7)

#### Theme 3: learning to understand the child across communication barriers

Communication barriers created a different kind of strain. Especially in the earlier stages of caregiving, mothers struggled to identify the child’s needs, emotions, or reasons for distress. When children could not express basic needs verbally, mothers relied on repeated attempts and subtle behavioural cues, learning through trial and error.

“There was a gap in understanding my child.” (P1) “I did not know how to communicate with him.” (P2)

This process sometimes created a painful sense of distance. Mothers described feeling helpless when their attempts to connect or understand did not succeed, although their accounts also suggested that they became more familiar with the child’s cues over time. Communication therefore emerged not as a fixed difficulty but as a relational learning process. Mothers gradually developed an informal repertoire of cues and trial-and-error strategies, suggesting that caregiving competence was built through repeated interpretation of the child’s behaviour rather than through verbal exchange alone.

“Communication required many attempts.” (P4) “I felt helpless when communication failed.” (P8)

#### Theme 4: the cumulative toll of continuous care

These demands accumulated into broader physical and emotional exhaustion. Mothers described constant supervision, repeated efforts to manage behaviour, and limited opportunities for rest. Sustained vigilance, particularly around safety, made uninterrupted sleep and genuine time away difficult.

“I felt emotionally and physically exhausted.” (P1) “Stress became part of my daily life.” (P2)

Several mothers described caregiving as reshaping the whole of daily life. Guilt could accompany even brief periods of time for themselves, making rest psychologically difficult as well as practically scarce. The burden was therefore cumulative rather than episodic: practical supervision, emotional vigilance, and guilt about stepping away compressed mothers’ opportunities for recovery. Continuous care became not simply a set of tasks, but an organising condition of everyday life.

“Caregiving drained my energy.” (P5; son aged 2–4 years, reported severe) “There was no time to rest.” (P7)

Cross-cutting influences: adaptation and family support.

#### Theme 5: adaptation through acceptance, patience, and faith

Over time, mothers described a gradual shift from initial distress towards adaptation. Patience, acceptance, learning from professionals, repeated attempts, and adjusted expectations helped them manage everyday challenges. They did not present acceptance as a single endpoint. Instead, it emerged gradually as they changed how they understood the child and their own caregiving role. For several mothers, faith formed an important part of this process, and some explicitly reframed autism as a spectrum or disorder rather than an illness.

“With time, I learned how to deal with him.” (P1) “I became more patient.” (P2)

Some mothers also described self-directed strategies, including structured routines and elimination diets that they had tried independently. These accounts document mothers’ coping practices and do not establish clinical effectiveness for the diets or other self-directed strategies.

“Acceptance made things easier.” (P6) “I adjusted my expectations.” (P7)

#### Theme 6: family support as a buffer and lack of support as an added burden

Family support often influenced how manageable caregiving felt. Mothers described spousal cooperation and help from extended family as reducing practical and emotional pressure through shared care, financial assistance, encouragement, and acceptance of the child. By contrast, mothers who received less support, or whose husbands denied the diagnosis, described carrying a larger share of the responsibility alone.

“My husband supported me a lot.” (P1) “Without family help, it would be impossible.” (P6; daughter aged 5–7 years, reported moderate)

### Domain 3: service access

#### Theme 7: compensating for uneven service experiences: cost, access, and advocacy

Mothers portrayed their service experiences as a structural backdrop to many of their accounts. They often became researchers, coordinators, and advocates for their children as they tried to bridge fragmented, costly, or poorly matched services. Cost emerged as the most concrete barrier. Mothers often described behaviour-modification specialists as private and expensive, even where the state funded centre-based care.

“The costs are very high: fifteen hundred a week, and five hundred for a short session; many centres take no insurance.” (a mother of a toddler)

Other accounts focused on limited follow-up, long waits that distressed the child, or environments that did not suit the child’s needs. Service-related difficulties also extended into education. In one account, peers bullied a child after a specialist disclosed the diagnosis at school.

“A specialist announced his diagnosis to the class and the children bullied him, so I moved him to another school.” (a mother of an older child)

The accounts were not uniformly negative. One mother described comprehensive state support, including free centres, transport, and a monthly payment. This counter-case sharpened the contrast with mothers who had to compensate for gaps in provision. Across the interviews, mothers called for greater community awareness, better teacher preparation, more affordable services, insurance coverage, priority arrangements for children who could not tolerate long waits, and clearer educational pathways into higher education.

“There are no costs; the state provides free centres, transport, and a monthly payment.” (a mother whose care was fully funded)

### Summary of themes

Taken together, the seven themes, grouped under the three domains of emotional distress, caregiving burden, and service access, show how emotional adjustment, everyday caregiving demands, family support, and service conditions shaped mothers’ experiences over time. For many mothers, diagnosis brought intense distress, whilst behavioural and communication challenges contributed to cumulative exhaustion. Acceptance, faith, and family support helped some mothers adapt. At the same time, service conditions cut across these experiences, either increasing mothers’ compensatory work or, in one counter-case, reducing it substantially. **Figure 1** summarises these relationships. Across these themes, maternal burden accumulated through overlapping forms of work: interpreting children’s needs, sustaining disrupted routines, regulating emotional responses, and coordinating family and service support. Adaptation reflected how mothers learned to navigate this work over time rather than the disappearance of burden.

**Figure 1:**
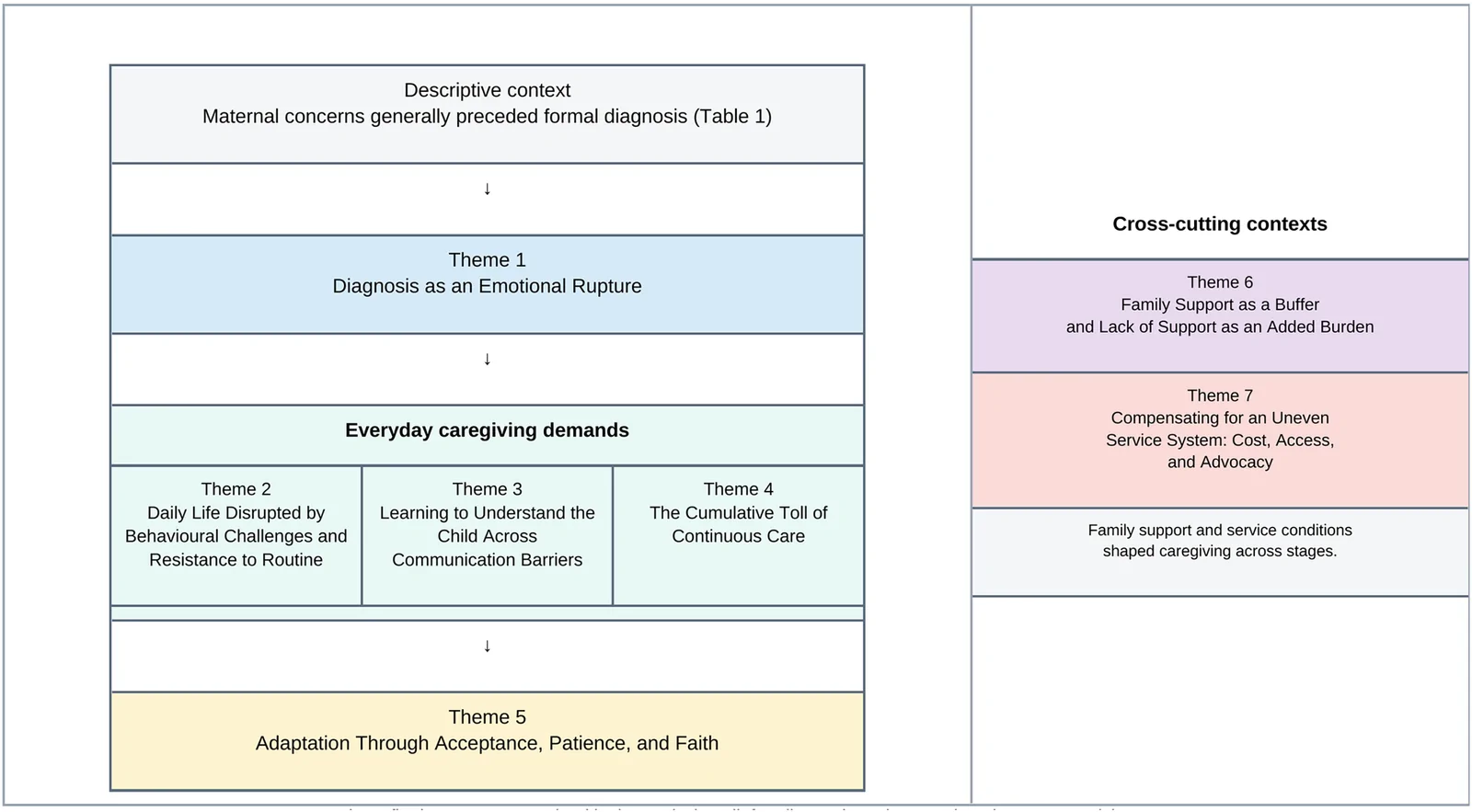
Thematic map of mothers’ experiences of raising a child with autism spectrum disorder. Maternal concern before formal diagnosis appears as descriptive context from **Table 1** rather than as a separate theme. Themes 1 to 4 capture the emotional and everyday caregiving burden; Theme 5 reflects adaptation over time; Theme 6 shows how family support can buffer caregiving demands or add to them when support is lacking; and Theme 7 represents the wider service context of access, cost, coordination, and advocacy. The analysis retained disconfirming accounts, including relief at diagnosis and comprehensive state provision. The three overarching domains of emotional distress, caregiving burden, and service access organise these themes, and family support and adaptation operate as cross-cutting influences.

## Discussion

This study explored how ten mothers in Madinah experienced raising a child with ASD. Their accounts did not point to a single dominant challenge. Instead, they described interconnected processes: diagnosis disrupted many mothers emotionally; behavioural and communication demands contributed to cumulative exhaustion; adaptation developed gradually through acceptance and faith; family support altered how manageable caregiving felt; and service gaps often required mothers to advocate for their children and coordinate care. From a public mental health perspective, these accounts show how maternal emotional wellbeing is intertwined with caregiving demands, family support, stigma, and service access. The findings broadly align with regional and international evidence whilst adding detail about how these processes converged in the Madinah context.

Participant characteristics also show that maternal concerns generally preceded formal diagnosis, sometimes by several years (**Table 1**). This pattern aligns with evidence that parents often notice developmental differences well before diagnosis (15–17). The present data do not establish why these intervals occurred. The analysis does not attribute the timing to inadequate services, provider knowledge, or systemic barriers; it reports the intervals as mothers described them. Previous Saudi research has documented broader gaps in autism-related services and support (18, 19). Those studies provide relevant context, but the present study did not test service factors as mechanisms of diagnostic delay.

Because the sample was small and recruited from one centre and the surrounding community, the analysis did not support systematic comparison across subgroups. No clear patterning by child sex, age band, or reported severity emerged in mothers’ accounts of distress, caregiving burden, or service access, and these experiences appeared across the sample rather than clustering in any one group. **Table 1** therefore offers context for the narratives rather than a basis for subgroup claims.

For most mothers, diagnosis marked an emotional rupture. The shock, sadness, anxiety, and physical effects they described align with the psychological burden reported amongst Saudi parents (10) and in broader reviews of caregivers of children with ASD (12). Yet mothers did not respond uniformly. One mother experienced relief because the diagnosis made her child’s behaviour more understandable. This counter-case matters because it shows that diagnosis can represent both the loss of an imagined future and an explanation that reduces uncertainty. These descriptions capture emotional distress as mothers reported it rather than clinically assessed disorder.

Emotional strain also entered family relationships. Some mothers supported husbands who struggled to accept the diagnosis or participated less in care, whereas supportive spouses and extended family reduced both practical and emotional strain. This pattern resonates with evidence of gendered differences in parental burden and coping (14, 26). Qualitative studies from Jordan, Iran, Greece, and the United Arab Emirates likewise show how family expectations, stigma, and social support shape caregiving (23, 24, 28, 29). This convergence helps contextualise the Madinah accounts, but it does not mean that families across these settings share a universal experience.

Within this study, acceptance appeared less as a final state than as a gradual shift in how mothers understood both the child and their own caregiving role. For several mothers, faith supported that shift, echoing the prominence of religious coping reported amongst Arab parents (8) and Muslim mothers in other settings (25). Mothers also described self-directed strategies, including elimination diets, similar to complementary practices reported amongst Arabic-speaking mothers elsewhere (36). Together, these accounts suggest that adaptation involved active meaning-making and experimentation rather than clinical input alone. They do not establish that acceptance, faith, or any particular coping strategy causes better outcomes.

In this sample, caregiving was embedded within intersecting family, religious, and service contexts. Extended-family relationships could either distribute or intensify the burden of care, whilst faith provided some mothers with a framework for acceptance and meaning-making. At the same time, considerable variation in mothers’ experiences of state-funded and privately accessed services cautions against treating the Saudi context as homogeneous. These findings therefore suggest that maternal adaptation in Madinah was shaped not only by individual coping but by the family and service environments within which caregiving occurred.

The study’s most distinct contribution lies in mothers’ accounts of compensating for uneven service experiences. They often located services, coordinated care, managed costs, and advocated for their children when they encountered fragmented or poorly matched provision. This finding aligns with reported service-related needs and variable service quality in Saudi Arabia (18, 19) and with low parental satisfaction with several support and intervention services (27). Stigma also entered institutional settings. One mother reported that a specialist disclosed her child’s diagnosis at school and that peers subsequently bullied the child, echoing Saudi research on discrimination and stigma (11, 22). By contrast, the counter-case of comprehensive state support showed that mothers did not inevitably have to perform this compensatory work. The amount of work appeared to expand or contract with the accessibility and organisation of services. Overall, these accounts show that uneven service experiences could generate an additional layer of maternal work: mothers became de facto navigators, coordinators, and advocates when formal provision was fragmented or poorly matched. The counter-case suggests that this compensatory work was contingent on service organisation rather than an inherent feature of mothering a child with ASD.

Finally, the findings support a more coordinated public mental health response to caregiver wellbeing. Psychological support around diagnosis and during follow-up could address the emotional strain mothers described, whilst practical guidance on communication, behaviour, and daily routines may reduce the trial-and-error burden of caregiving. At service level, the accounts support attention to affordability, insurance coverage, continuity of care, and waiting arrangements that work for children with ASD (19, 27). The bullying account also supports school-based autism education and stronger safeguards around disclosure, consistent with wider research on stigma (11, 22). When considered alongside previous research, the findings reinforce the value of autism-related knowledge amongst frontline professionals, accessible referral pathways, and caregiver mental health support as linked elements of care provision. These priorities are consistent with Vision 2030’s broader quality-of-life goals (31).

The study has several limitations. It recruited ten mothers through one autism centre and the surrounding community in Madinah, so it does not capture the perspectives of fathers, children, or mothers who remain disconnected from formal services. Recruitment through a centre and a self-selected online form may also have favoured mothers who engaged more actively with care or resource-seeking, which may have made advocacy more visible in the findings. Mothers gave retrospective, self-reported accounts, and translation from Arabic to English may have altered some nuance despite bilingual checking. The sample and context therefore bound transferability, rather than sample size alone. Future research could include fathers and, where appropriate, children, recruit across socioeconomic and service-access settings, and examine how experiences change with the child’s age and time since diagnosis. Only a minority of participants were connected to the recruiting centre, whether by receiving care there or, in one case, working there, so a few mothers may have been cautious about criticising its services; because most participants used services elsewhere, this social-desirability risk was probably limited.

## Conclusion

This qualitative study examined how ten mothers in Madinah experienced raising a child with ASD. Three findings stand out. First, mothers generally noticed concerns before formal diagnosis, and diagnosis often brought intense shock, sadness, and worry that continued into everyday caregiving. Second, mothers described gradual adaptation through acceptance, patience, faith, and family support, although these resources were not equally available to everyone. Third, many mothers compensated for fragmented, costly, or poorly matched services by advocating for their children and coordinating care. The counter-case of comprehensive state provision illustrated a markedly different experience when coordinated support was available. From a public mental health perspective, maternal wellbeing should be understood in relation to the family resources and service conditions within which caregiving occurs. The findings therefore position maternal resilience not as an individual solution to inadequate support, but as a response shaped by family relationships, cultural resources, and service structures.

### Implications and recommendations

The findings suggest several practical priorities. Around diagnosis, mothers may benefit from accessible psychological support and clear, family-centred guidance on communication, behaviour, and daily routines. Nurses and other frontline professionals can help by linking families to appropriate services and reinforcing consistent information. Services should also engage fathers and extended family when the mother considers this appropriate. At policy level, mothers’ accounts support attention to affordability, insurance coverage, continuity of follow-up, and priority arrangements for children who find prolonged waits difficult. Teacher preparation and school-based autism awareness can help reduce stigma, whilst clear procedures should protect confidentiality. Mothers also raised the need for an educational pathway that extends to higher education, which warrants further policy attention. Future research should include fathers and, where appropriate, children, recruit across socioeconomic and service-access settings, and follow families longitudinally to examine how coping, family support, and service conditions shape adaptation over time. These priorities are also consistent with Vision 2030’s broader quality-of-life goals (31).

## Data Availability

The raw data supporting the conclusions of this article will be made available by the authors, without undue reservation.
